# Extramedullary hemopoiesis with undiagnosed, early myelofibrosis causing spastic compressive myelopathy: Case report and review

**DOI:** 10.4103/0019-5413.57281

**Published:** 2010

**Authors:** Udita Dewan, Niraj Kumari, Awadesh Jaiswal, Sanjay Behari, Manoj Jain

**Affiliations:** Department of Pathology, Sanjay Gandhi Postgraduate Institute of Medical Sciences, Lucknow, India; 1 Department of Neurosurgery, Sanjay Gandhi Postgraduate Institute of Medical Sciences, Lucknow, India

**Keywords:** Extramedullary hemopoiesis, myelofibrosis, spastic paraparesis, spine

## Abstract

Extramedullary hemopoiesis (EMH) is a common compensatory phenomenon associated with chronic hemolytic anemia. Abnormal hemopoietic tissue usually develops in sites responsible for fetal hemopoiesis, such as spleen, liver and kidney; however, other regions such as the spine may also become involved. In this study, a patient presenting with spastic paraparesis due to EMH in the dorsal spine is described. A 62-year-old man presented with paraparesis. Magnetic resonance imaging revealed a large lesion involving the T2-L2 vertebral levels with a large extradural component causing thecal sac compression. Laminectomy with excision of mass was carried out. The histopathology revealed EMH. The patient had no known cause for EMH at the time of diagnosis but, subsequently, a bone marrow examination revealed early myelofibrosis. This case represents the rare occurrence of a large extradural extramedullary hematopoiesis in a patient with no known predisposing factor for hemopoiesis at the time of presentation.

## INTRODUCTION

Extramedullary hemopoiesis (EMH) may occur in various types of hemodyscrasia and dyshemopoiesis as a common compensatory phenomenon associated with chronic hemolytic anemia.^1^,^2^ Abnormal hemopoietic tissue usually develops in sites responsible for fetal hemopoiesis, such as spleen, liver, lymph nodes and kidney; however, other regions such as the spine may also become involved.^1^,^2^ In patients with thalassemia and other hemoproliferative disorders, EMH may occasionally lead to progressive spastic myelopathy.^1^–^3^ In the present study, however, spinal thecal and cord compression as a consequence of EMH in the intraspinal epidural space initially manifested without any primary cause. Asymptomatic myelofibrosis in the early stages was subsequently detected on bone marrow examination. In this study, a patient presenting with spastic paraparesis due to EMH in the dorsal spine has been described.

## CASE REPORT

A 62-year-old male patient presented with progressive, spastic paraparesis over a period of 4 months with complaints of tightness in the bilateral lower limbs, progressive difficulty in walking and band-like sensation at the level of the nipples. He complained of numbness of the bilateral lower limbs, frequency of micturition and constipation for 1 month. He had a history of cervical lymphadenopathy 2 years back for which he took antituberculous treatment (isoniazid, rifamycin, pyrizinamide and ethambutol) for 9 months. He was a known hypertensive for the last 9 months.

His general physical and systemic examinations were unremarkable. On neurological examination, his higher mental functions and cranial nerve examination were normal. The motor system examination revealed spastic paraparesis with Medical Research Council grade 0 at the hip and knee and grade 2 movement at the ankle and toes. Superficial abdominal reflexes were absent and bilateral plantar reflexes were extensors. The sensory examination revealed 50–70% sensory loss of all modalities below the T4 level, including posterior column sensation impairment in both lower limbs. A diffuse spinal tenderness was present in the mid dorsal spine and the flexion–extension movements of the dorsal spine were restricted and painful. The clinical impression was that of an extradural compressive myelopathy with T4 spinal cord level.

His routine blood investigations revealed hemoglobin of 10.3 gm% and total leukocyte count of 9,300 cells/mm^3^, with differential leukocyte count of neutrophils (71%) and lymphocytes (29%). A few nucleated red blood cells (RBCs) were noted in the peripheral smear along with findings of anisopoikilocytosis, few tear drop cells and polychromatophilic RBCs.

**Figure 1:**
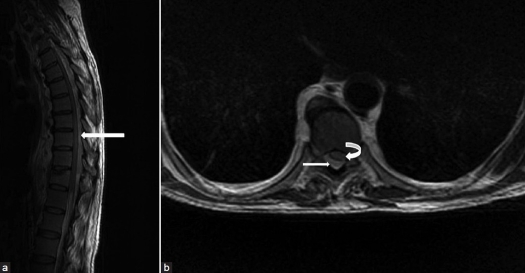
(a) T2-weighted sagittal magnetic resonance image of the thoracolumbar spine showing the isointense extradural lesion (arrow) causing thecal sac and spinal cord compression and signal intensity changes within the cord. (b) The T2-weighted axial image showing thecal and spinal cord compression (curved arrow) by the extradural lesion (straight arrow)

A magnetic resonance imaging (MRI) of the dorsal spine revealed a large lesion involving the T2-L2 vertebral levels. The lesion was hypointense on T1-and isointense on T2-weighted images with a large extradural component. It caused thecal sac and spinal cord compression with T2-signal intensity changes within the cord [Figure 1a and b]. There was no evidence of caseation or necrosis within the lesion. Plain radiographs were not performed before surgery.

The patient was planned for a T2-L2 laminectomy with excision of the mass. During surgery, the epidural veins were engorged. The veins and the mass bled profusely during surgical dissection. The extradural lesion was soft, pinkish and friable. A peroperative impression of lymphoma was considered. The patient was anemic in the post-operative course, with the post-operative hemoglobin being 6.4 gm%. Multiple blood transfusions and oral hematinics were administered. The patient was discharged on the seventh post-operative day.

**Figure 2:**
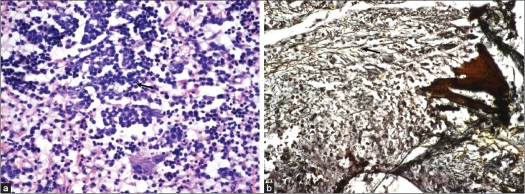
(a) Myeloid cells (arrow) admixed with erythroid cells and megakaryocytes (H and E, ×40). (b) Increased reticulin (arrow) on bone marrow biopsy (Retic ×40)

The histopathology of the lesion showed a vaguely nodular collection of hematopoietic cells comprising of various stages of erythroid cells, myeloid cells and megakaryocytes with an intertraversing sinusoidal capillary network [Figure 2a]. A histolological confirmation was obtained by staining these sections with periodic acid Schiff stain for megakaryocytes and chloroacetate esterase for myeloid cells, respectively. The megakaryocytes showed magenta-colored cytoplasmic positivity with periodic acid Schiff stain and the myeloid cells showed brownish, granular cytoplasmic positivity with chloroacetate staining.

A diagnosis of extramedullary hematopoeisis was established on the basis of characteristic morphology of hematopoietic cells and their confirmation after histochemical stains. A bone marrow examination was carried out to ascertain the cause of anemia and extramedullary hematopoeisis. The bone marrow biopsy revealed hypercellular marrow with erythroid hyperplasia and megakaryocytic clustering with ectatic sinusoids. The megakaryocytes showed evidence of dysplasia manifested by nucleomegaly, coarse chromatin and absence of nuclear lobation. Reticulin staining showed focally increased fibrosis [Figure 2b]. These findings were suggestive of early myelofibrosis and a close follow-up was advised. On follow-up after 2 months, a Technetium 99 colloid scan and denatured RBC nuclear scan of the liver and spleen were performed, which revealed normal liver and spleen scan findings with no evidence of extramedullary hematopoeisis in these organs. At follow-up after 1.5 months, the patient showed grade 3 power in both lower limbs with residual spasticity. He did not report for a subsequent follow-up and did not undergo a repeat MRI.

## DISCUSSION

EMH refers to the presence of hemopoietic elements in locations other than the bone marrow medullary space. It may be seen in many conditions, including chronic anemias, blood dyscrasias such as leukemia or as an incidental finding. The common sites of involvement are the liver, spleen and the lymph nodes. Less common sites include the central nervous system, adrenal gland, kidney, perirenal soft tissues, breast, peritoneal surfaces and gastrointestinal tract.^4^–^7^ The paraspinal region is a relatively uncommon location for these hemopoietic deposits. There are a few case reports of EMH occurring in the spine.^1^–^3^,^8^ Most cases have been reported in connection with thalassemia, although it has also been described in patients with sickle cell anemia, myelofibrosis and polycythemia vera and myelodysplastic syndrome.^9^–^11^ To the best of our knowledge, there are very few documented case reports in the literature of patients with myelofibrosis who developed cord compressive symptoms due to EMH in the spine.^8^,^10^ The patients had myelofibrosis of almost 10 years duration. We report the rare case of a 62-year-old male patient who was diagnosed with compressive symptoms due to solitary EMH in the epidural space. At the time of the appearance of his clinical manifestations due to EMH, he did not have any manifestations of the primary disease that could be responsible for the development of his EMH. The diagnosis of the coexisting, early stages of myelofibrosis (that was essentially asymptomatic) could only be established on subsequent bone marrow examination. There was no evidence of EMH in the liver and spleen in our patient. In all the other reported cases of EMH secondary to myelofibrosis, the manifestations of myelofibrosis precede the development of EMH by several decades.

The source of the epidural hemopoietic tissue in EMH is controversial. There may be rests of primitive hemopoietic stem cells that later expand under extreme hematologic stress present in patients with severe chronic anemia; or, blood-forming elements in the vertebral marrow may be extruded through weakened trabecular bone into the epidural compartment where they may proliferate. Epidural lymphoma, metastases and tuberculosis may show a similar picture.

MRI and nuclear scans delineate ectopic foci of hemopoietic tissue in addition to precisely defining the location, size and extent of the lesion. In our case, however, EMH could not be detected at any other site.

Idiopathic myelofibrosis (IMF) implies fibrosis of the bone marrow in the absence of any associated disease. The average age at diagnosis is 60 years. Splenomegaly due to EMH is a distinctive feature of IMF. The splenomegaly is secondary to myeloid metaplasia. The degree of splenic hemopoiesis has been shown to be directly associated with the duration of the disease.^12^ In a study by Andreasan, 13 of 56 cases of IMF did not have splenomegaly.^13^ Six of 100 patients with IMF reviewed by Bouroncle and Doan did not have splenomegaly at the time of diagnosis.^14^ They stated that during the course of the disease, progressive enlargement of the spleen is a rule. Pitcock *et al*. noted that two of 70 patients did not have splenomegaly at the time of diagnosis of IMF.^15^ No follow-up of these patients without splenomegaly has been mentioned in these reports. There has been only one case report of a patient with IMF of 8 years duration with lack of splenomegaly. In the present reported case, the patient may have been in the stage of early fibrosis, which can explain the absence of splenomegaly and anemia at the time of diagnosis. Even 2 months after the diagnosis, no evidence of EMH or splenomegaly was found by nuclear scan and imaging.

No definite guidelines have as yet been formulated for the treatment of such patients with EMH. Excision and decompression, radiotherapy, hypertransfusion and hydroxyurea alone or in combination have been proposed. These lesions are extremely vascular (as seen in our patient) and hemorrhage in patients with chronic anemia has several risks of cardiovascular debility. Incomplete resection and a high incidence of recurrence are also frequently observed. Radiotherapy has shown a good response in a recent review of spinal EMH.^16^

The reported patients in the literature are summarized in Table given as additional material on http://www.ijoonline.com/viewimage.asp?img=IndianJOrthop_2010_44_1_98_57281_t3.jpg. The most common causes of EMH included thalassemia, polycytemia, hereditary spherocytosis, sickle cell anemia and myelofibrosis. According to the literature, both surgery and radiotherapy have been administered with reasonable results, although recurrences may occur.^3^,^17^–^69^ In our patient, myelofibrosis was asymptomatic and there was no anemia. Therefore, extradural long-segment EMH may have been the primary pathology associated with early myelofibrosis and was not consequent to the anemia produced by myelofibrosis, as has usually been reported in the literature.

To conclude, the unique findings of extensive, solitary EMH of the spine leading to spastic paraparesis with coexistence of early stages of asymptomatic myelofibrosis and the absence of anemia may point toward the rare possibility that EMH and myelofibrosis in our patient had a concurrent association and that EMH had not developed as a compensatory phenomenon consequent to myelofibrosis.
